# Electric-Field-Coupled Resonator Antenna for 5G Applications

**DOI:** 10.3390/ma15155247

**Published:** 2022-07-29

**Authors:** Md. Mushfiqur Rahman, Md. Shabiul Islam, Mohammad Tariqul Islam, Touhidul Alam

**Affiliations:** 1 Faculty of Engineering, Multimedia University, Persiaran Multimedia, Cyberjaya 63100, Selangor, Malaysia; 1181402135@student.mmu.edu.my; 2 Department of Electrical, Electronic & Systems Engineering, Faculty of Engineering and Built Environment, Universiti Kebangsaan Malaysia, Bangi 43600, Selangor, Malaysia; 3 Pusat Sains Ankasa (ANGKASA), Institut Perubahan Iklim, Universiti Kebangsaan Malaysia, Bangi 43600, Selangor, Malaysia; touhidul@ukm.edu.my

**Keywords:** LC resonator, capacitive gap, parasitic element, wideband, omnidirectional radiation pattern, compact

## Abstract

In this paper, a compact wideband patch antenna comprising a modified electric-field-coupled resonator with parasitic elements is presented. The resonance at low frequency is achieved due to the electric field polarization along the split of the conventional LC (inductive-capacitive) structure. However, this antenna gives low bandwidth as well as low gain. Some evolutionary techniques are adopted to get a compact wideband antenna at 3GPP bands of 5G. The split width and the ground plane are modified to achieve enhanced bandwidth with good impedance matching, whereas the addition of the parasitic elements on both sides of the microstrip feed line enhances the gain with a slight reduction of bandwidth. The compact dimension of the proposed antenna is 0.26 λ_L_ × 0.26 λ_L_ × 0.017 λ_L,_ where λ_L_ is the free space wavelength at the lowest frequency. A prototype of the presented design is fabricated and measured. Measurement shows that the antenna has an operating bandwidth of 19.74% for |S11| < −10 dB where the gain of 1.15 dBi is realized. In addition, the radiation pattern is omnidirectional in the horizontal plane and dumbbell shaped in the elevation plane. The cross-polarization levels in both planes are less than −12 dB.

## 1. Introduction

The development of tiny devices with the advancement of technology is in high demand. Connecting these devices with a wireless network is also mandatory to control them from remote places. Especially, the latest 5G technology will ensure massive IoT deployment in the industry with high speed and low latency. For this reason, compact and wideband antenna design is one of the most important fields of research in this modern era. A compact antenna can be designed by increasing the electrical length of the antenna so that it can give resonance without changing the overall dimension of the antenna.

In recent research, various techniques have been applied to increase the electrical length to fulfill the half-wavelength principle of resonance. The techniques can be divided into two categories. One category utilizes the techniques of cutting slots, the techniques of using various shapes, or the combination of these two. For example, a Y-shaped slot tapered in a printed dipole results in a multi-band compact antenna [1]. Two T-shaped patches are placed back-to-back to get an omnidirectional radiation pattern, which is compact at 2.4 GHz [2]. A taper-shaped radiating element is presented which is electrically small at the center frequency of 2.88 GHz [3]. A loop-fed C-shaped structure with a small shorting-wall-connected ground plane is proposed to get a compact antenna but results in poor bandwidth [4]. Four centrally grounded parasitic patches are loaded into four corners of a meander-shaped ring patch to get a miniaturized radiator [5]. The combination of Koch fractal geometry and meandering slits is employed to increase the electrical length without the increment of patch area [6]. A fractal antenna with a hexagon-shaped nested loop is proposed which is compact at 1.7 GHz [7]. A low-profile antenna is proposed where the radiating patch is meandered with the use of an open-ended ground slot and shorted pin between the ground plane and the patch [8]. An ultra-miniaturized antenna is designed by creating rectangular slots in the ground and the patch. The antenna shows good bandwidth but very low gain, which limits its applications in wireless communications [9]. In one example of a rectangle-shaped patch with a full ground plane, two U-shaped slots are cut on the patch and a slanted slot is cut on the ground plane to get a compact and wideband antenna. However, for high-frequency portable device applications, it is not compact enough [10]. Two triangle-shaped structures, with triangle-shaped slots cut on both of them, are connected together at the base by a rectangular block to make a patch of a compact antenna, where the bandwidth is reported to be very poor [11].

The other category involves the partial loading of loop resonators (with single or multiple slits) with a particularly shaped resonator to improve the performance or to get a miniaturized structure. Namely, two square-circular split-ring resonator structures are placed in parallel and loaded on the main patch of the antenna. Here, the ring resonators are used to get low-frequency resonance [12]. A penta-ring antenna is presented to get multiband characteristics, which are electrically small at 2.2 GHz, and the impedance bandwidth is 500 MHz of that band [13]. A complimentary split-ring resonator is loaded into a semicircular monopole antenna to achieve multiband operation which is compact at 1.7 GHz [14]. A split-ring resonator and a complementary split-ring resonator are interconnected to a patch to get a multiband property as well as a compact structure. Here, the capacitive gap is responsible for achieving low-frequency resonance [15]. Two split-ring resonator structures are interconnected to a meander line to get a compact structure [16]. A rectangle-shaped complementary split-ring resonator is connected inside a ring monopole to get multiband characteristics as well as a compact structure [17]. A triangle-shaped structure is engraved with two rectangle-shaped slots and a triangular split-ring resonator to achieve a multiband and compact antenna. This antenna exhibits poor impedance matching at low frequency as well as poor bandwidth and gain [18].

In all the literature mentioned here, compactness is achieved by using the substrates of high dielectric values. According to the half-wavelength principle of resonance, materials of higher dielectric constants will cause small dimensions, but they will account for more dielectric loss. However, for the application of 5G, the communication needs to be more efficient, which requires the utilization of low dielectric constant materials in antenna design. For this reason, the proposed structure used the substrate of a low dielectric constant, where achieving compact dimension is a challenge. However, there are some special cases where the substrate is chosen depending on the demand of the application. For instance, reference [8] used Rogers 6010, which is suitable for microelectronics, and reference [9] used Rogers ULTRALAM, which is suitable for implantable device applications as it is biocompatible. In addition, all of them except reference [3] give narrow bandwidth at the low-frequency band. So, compact and wideband antenna design for the latest generation of technology needs to utilize the materials of low dielectric constants to ensure low loss communication.

In this paper, an electric-field-coupled resonator is used as the main radiator of the antenna rather than partially loaded with different shaped patches. With these types of resonators, compactness can be achieved by using the substrates of low dielectric constants. This is possible due to the fact that the resonance of these structures depends on the loop inductance and gap capacitance. So, the requirement of the half-wavelength principle does not need to be fulfilled [19,20]. However, the drawback is that it shows very poor performance, i.e., narrow bandwidth and low gain. Here, the patch and the ground plane are modified to obtain enhanced impedance bandwidth, and parasitic elements are added to increase the gain without changing the dimension of the antenna.

## 2. Antenna Design and Analysis

**Figure 1:**
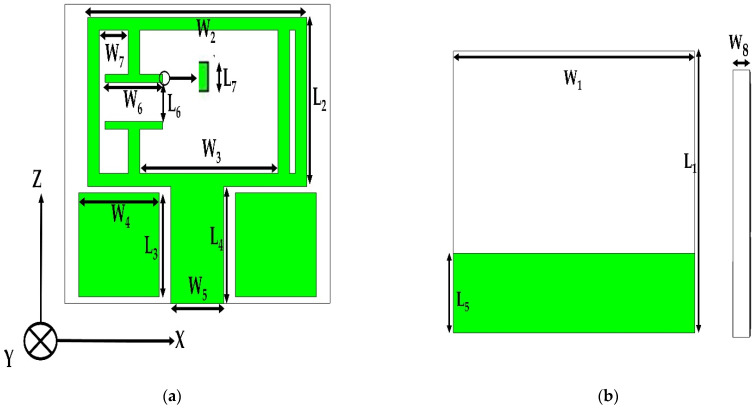
The configuration of the proposed antenna. (**a**) Top view; (**b**) Bottom view; and (**c**) Side view.

RT Duroid 5880 substrate of dielectric constant 2.2 and loss tangent 0.0009 with a thickness of 1.52 mm is used as the dielectric material of the antenna. The final design of the antenna is shown in Figure 1, and its dimensions are listed in Table 1. The design of the antenna is started by the analysis of the ELC (electric LC) resonator with appropriate boundary conditions. Then, the initial antenna is formed by exciting the resonator by a microstrip feed line with a finite ground plane size.

Further, some evolutionary steps are applied to enhance the performance of the antenna. A detailed description of the entire analysis is given in the subsequent sections.

## 3. Modelling of the ELC Resonator

The length and width of the ELC resonator are 13 mm and 19 mm, respectively, while the thickness of each side is 1 mm. The length and width of each arm of the capacitive gap are 5 mm and 0.6 mm, respectively, while the gap distance is 0.6 mm. The substrate material used here is RT Duroid 5880 with a dielectric constant of 2.2 and a thickness of 1.52 mm.

Since the resonator is anisotropic, i.e., it shows different resonance characteristics for different applied field orientations, it needs to be investigated for different boundary conditions [21,22,23,24]. However, the boundary conditions where the magnetic field is perpendicular to the resonator are not discussed here. This is because the symmetry of the ELC structure belongs to the C2h group, which does not possess magnetoelectric coupling [25].

**Figure 2:**
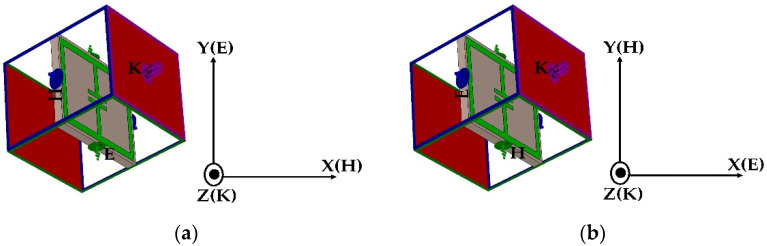
Simulation setup of the ELC unit cell. (**a**) Setup 1; (**b**) Setup 2.

For this reason, the presented structure is modeled by two boundary conditions. In one boundary condition, the electric field is applied along the y axis, and the magnetic field is applied along the x axis (Setup 1), whereas in another boundary condition the field orientations are opposite to the former one (Setup 2) (see Figure 2).

**Figure 3:**
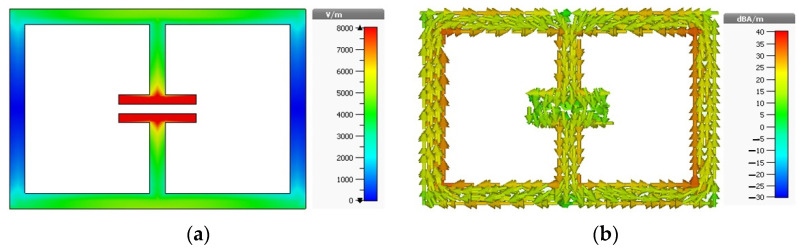
Distribution of (**a**) electric field and (**b**) surface current at 2.67 GHz for Setup 1.

**Figure 4:**
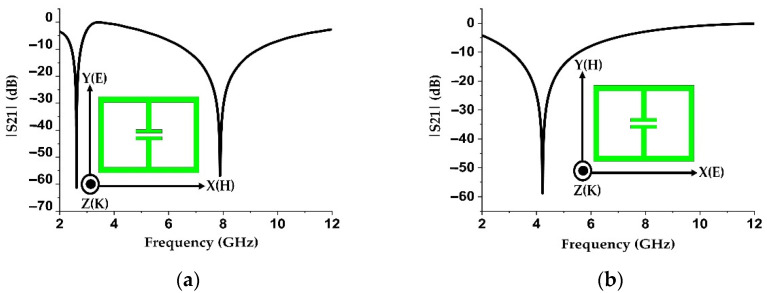
S21 spectrum of the ELC resonator for (**a**) Setup 1 and (**b**) Setup 2.

The resonator with these two setups is simulated using a time-domain solver using CST Microwave Studio software. Since in Setup 1 the electric field is polarized along the split (see Figure 3a), it induces a current in the structure that circulates in two loops as shown in Figure 3b, resulting in two different current paths. Due to these current paths, two resonances are found at 2.67 GHz and 8.03 GHz frequencies (see Figure 4a).

**Figure 5:**
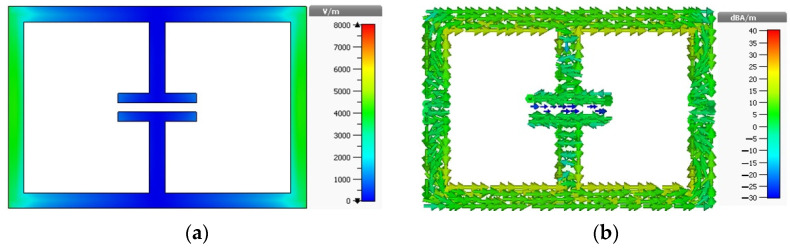
Distribution of (**a**) electric field and (**b**) current densities at 4.23 GHz for Setup 2.

On the other hand, when the electric field is polarized along the non-split bearing sides (see Figure 5a), the induced current is confined to the opposite sides of the resonator. For this reason, it cannot complete a loop and results in a reduced electrical length as shown in Figure 5b. For this reason, a single resonance occurs at 4.23 GHz (see Figure 4b), which is higher than the previous orientation (2.67 GHz).

## 4. Performance Analysis of the ELC Antenna

**Figure 6:**
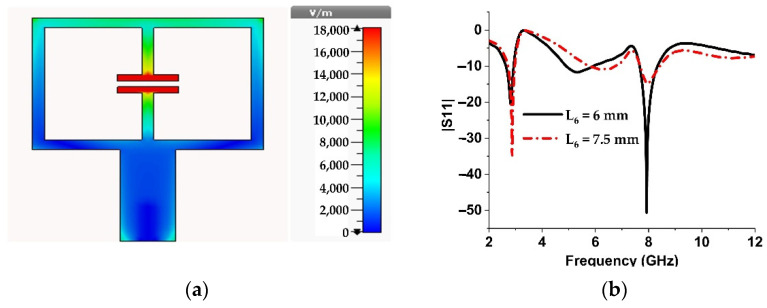
(**a**) Electric field distribution of Antenna 1. (**b**) Simulated resonance characteristics of Antenna 1 for different ground plane sizes.

From the above analysis, it is clear that when the resonator is used as the radiating element of an antenna, it will exhibit the same resonance characteristics provided the substrate material and its thickness, as well as their orientation, are the same for both cases. The same orientation can be achieved when the structure is excited by a microstrip feed line in a way so that the polarization of the electric field will be in the same direction. Since Setup 1 causes low-frequency resonance, the antenna will be miniaturized if the ELC is excited in the way shown in Figure 6a because then the electric field will be polarized along the capacitive gap as in Setup 1.

However, antenna simulation shows that when the ELC is excited as shown in Figure 6a, then two resonances occur at almost the same frequencies (2.88 GHz and 7.96 GHz) as in Setup 1, but they are matched with different ground plane sizes as depicted in Figure 6b. For compactness, the ground plane size L6 = 7.5 is chosen. However, this antenna results in a narrow bandwidth (10.41%) and poor gain (0.07 dBi).

**Figure 7:**
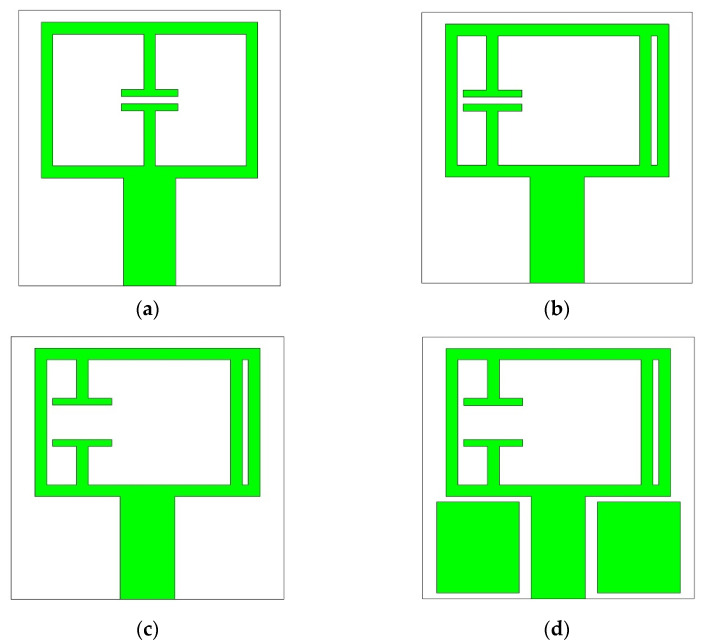
Patch of (**a**) Antenna 1; (**b**) Antenna 2; (**c**) Antenna 3; (**d**) Antenna 4.

The performance of Antenna 1 is improved at the desired frequency region by some evolutionary steps as shown in Figure 7.

Since the antenna is designed for the 5G C band applications, the resonant frequency needs to be shifted to the higher frequency region. It can be achieved by reducing the length and width, reducing the electrical length, or by decreasing the gap capacitance of Antenna 1. The first method is avoided since it can cause an even less efficient radiator. However, the second method is implemented in two steps.

**Figure 8:**
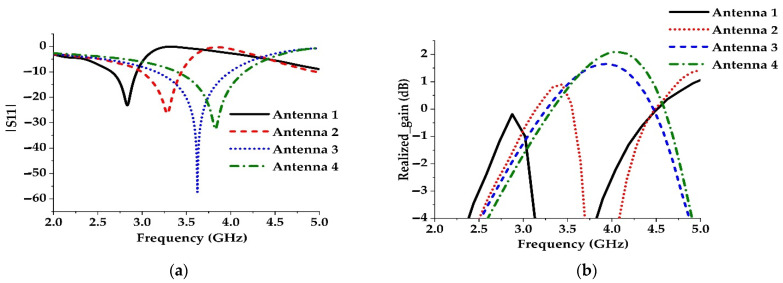
(**a**) Comparison of S-parameters of the antennas. (**b**) Comparison of gains of the antennas.

Firstly, the capacitive gap is moved near to the left side, which changes the current path inside the structure, and, secondly, an inductive strip is connected in parallel near to the right side of the structure (Antenna 2 in Figure 7b). As a result, the electrical length, responsible for the low-frequency resonance, is reduced, which shifts the resonance to the higher frequency region as shown in Figure 8a. However, the second method has a negligible effect on bandwidth, but the gain is increased considerably as shown in Figure 8b.

**Figure 9:**
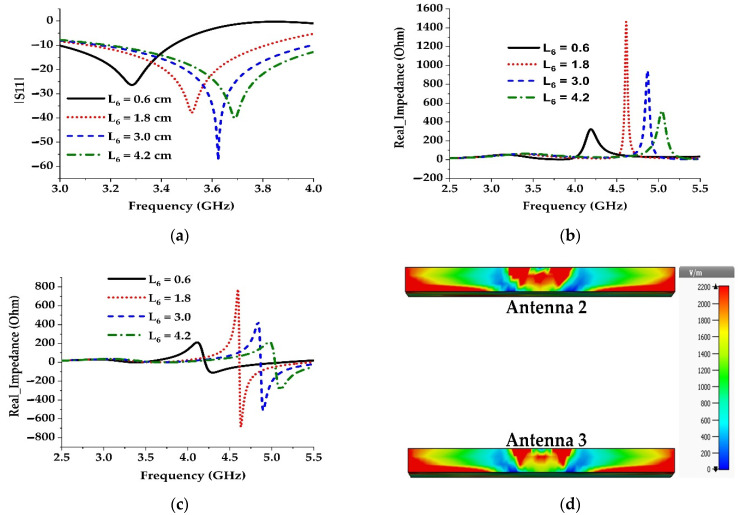
Variation of reflection coefficients and real and imaginary impedance values with different gap widths. (**a**) Reflection coefficients; (**b**) Real impedances; (**c**) Imaginary impedances; (**d**) Simulated electric field distribution in the cross-section of Antenna 3 and Antenna 4.

The third method is applied by increasing the capacitive gap width of the resonator (Antenna 3 in Figure 7c), which causes the gap capacitance to decrease. For this reason, the resonance is shifted to the higher frequency region (Figure 8a). A parametric study on the gap width is shown in Figure 9a to demonstrate the effect of gap capacitance on reflection coefficients. The study shows that increasing the capacitive gap shifts the resonance to the higher frequency region and increases the bandwidth as well.

However, the effect of gap width on antenna bandwidth can be easily realized by the parametric studies of various gap widths with real and imaginary values of the input impedance (see Figure 9b,c). As the gap width increases, the real impedance value decreases and the imaginary value flatters at high frequency, which causes a wide bandwidth. This happens because the electric field between the microstrip feed line and the ground plane decreases as the gap width increases, which in turn decreases the coupling capacitance between the feed and the ground, which is shown in Figure 9d. The optimized value of the gap width (L6) is chosen to be 3 mm. Further increment of the gap width results in poor impedance matching and the bandwidth enhancement is also negligible.

Finally, the gain of the antenna is improved by adding two rectangular metallic blocks of the same size on both sides of the microstrip feed line (Antenna 4 in Figure 7d). These parasitic elements increase the current density at the side near the capacitive gap and at the lower side of the structure, which causes the gain to increase as presented in Figure 8b. A slight reduction in bandwidth is also observed.

**Figure 10:**
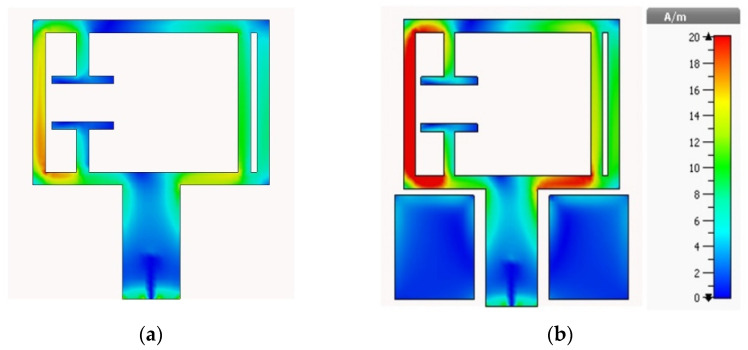
Surface current distribution of (**a**) Antenna 3 and (**b**) Antenna 4 at 3.6 GHz and 3.8 GHz, respectively.

The effect of adding the rectangular blocks can be easily detected from the surface current distribution of the antenna. As seen in Figure 10, the blocks increased the current densities on the overall patch, especially at the lower portion of the radiator and the left portion of the radiator near the gap. As a result, the gain of the antenna is considerably increased.

**Figure 11:**
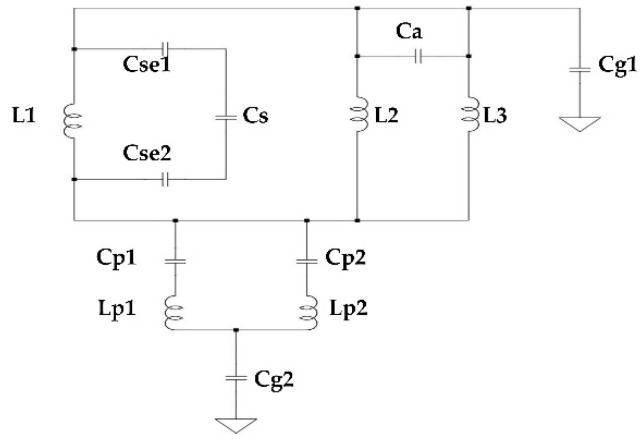
Equivalent circuit of the proposed antenna (Antenna 4 in Figure 7d). Here, Cse1 and Cse2 = The capacitance between the edges of the capacitive gap and the left arm. Cs = The capacitance due to the gap of L6. Ca = The capacitance be-tween the two arms on the right side. Cp1 and Cp2 = The capacitances between the parasitic blocks and the patch.Cg1 and Cg2 = The coupling capacitances between the patch and parasitic blocks with the ground.

The equivalent circuit of the structure can be approximated as shown in Figure 11.

## 5. Results and Discussion

**Figure 12:**
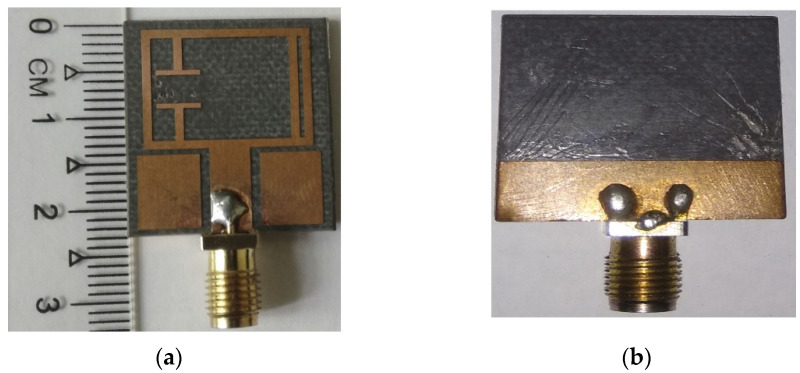
Fabricated (**a**) top view and (**b**) bottom view of the proposed antenna.

**Figure 13:**
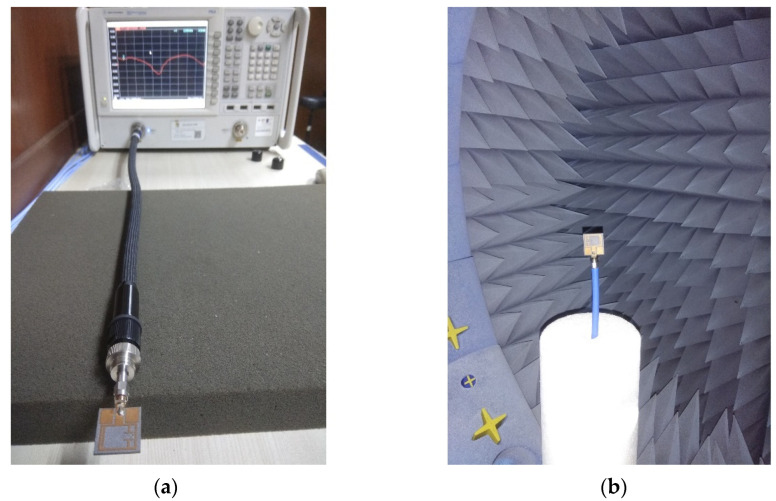
Measurement setup of (**a**) PNA and (**b**) Satimo Star Lab.

**Figure 14:**
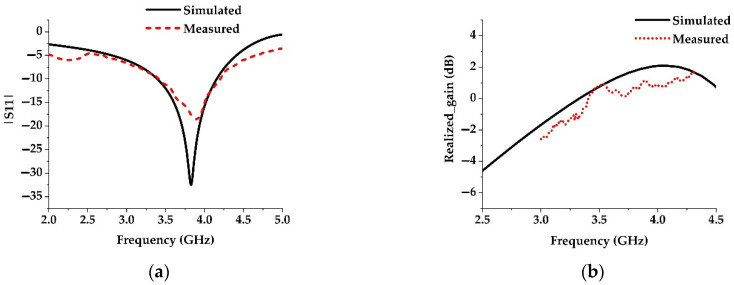
Simulated and measured (**a**) reflection coefficient and (**b**) realized gain of the proposed antenna.

A prototype of the proposed antenna (Antenna 4 in Figure 7d) is fabricated and measured to validate the above analysis. The simulated results are in good agreement with measured results, where a slight variation is observed. It is actually due to the cable loss and fabrication tolerance. In addition, the cable used in the Satimo StarLab measurement system is very long. The reflection coefficient of the prototype is measured using a PNA network analyzer N5227A (10 MHz to 67 GHz), whereas the radiation patterns and gain are measured using the Satimo StarLab antenna pattern measurement system. The photography of the fabricated prototype is presented in Figure 12. The measurement setup of PNA and Satimo Star LAB are shown in Figure 13a,b. The simulated and measured reflection coefficient and realized gain of the prototype are shown in Figure 14a and b, respectively. The measured reflection coefficient for |S11| < −10 dB is from 3.4 GHz to 4.2 GHz, and the maximum gain of 1.16 dBi is realized.

**Figure 15:**
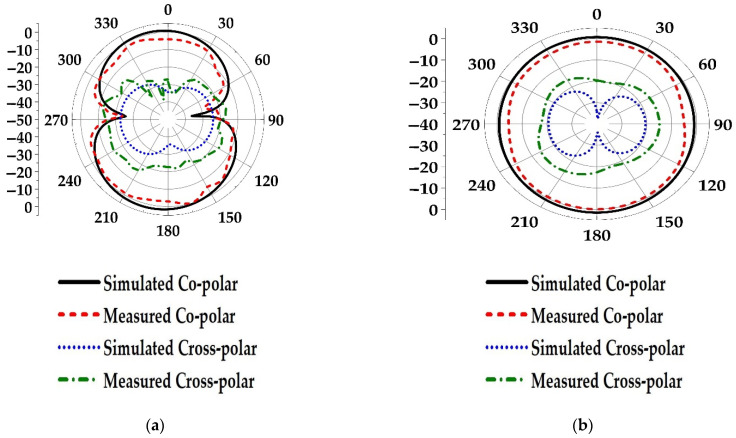
Simulated and measured radiation patterns of the proposed antenna. (**a**) YZ-plane (elevation plane), (**b**) XY-plane (horizontal plane).

**Figure 16:**
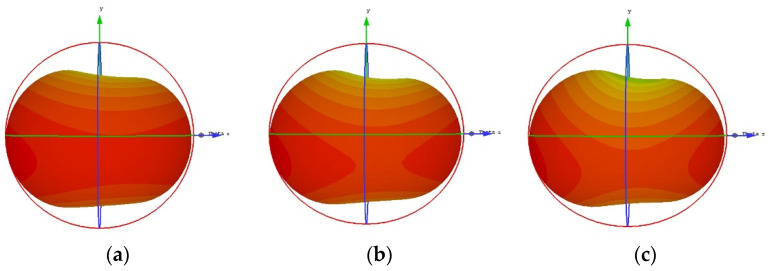
Simulated 3d gain at (**a**) 3.4 GHz, (**b**) 3.9 GHz, and (**c**) 4.2 GHz.

The simulated and measured radiation patterns in the elevation (E-plane) and horizontal plane (H-plane) are depicted in Figure 15a,b, respectively, which are in good agreement. The measured E-plane radiation pattern is dumbbell-shaped while the H-plane radiation pattern is omnidirectional, and both patterns are stable. However, the measured cross-polarization levels in both planes are less than −12 dB. The simulated 3d gain plots at three different frequencies are shown in Figure 16.

A comparison between the proposed antenna with some published referenced research is listed in Table 2. All the research works except reference [5] use substrate materials of high dielectric constants, which will cause high dielectric loss. They are slightly smaller or equal in electrical length to the proposed state-of-the-art. However, all of them have the problem of low bandwidth at the low-frequency band except references [3,9] and the presented work. However, reference [9] shows a huge difference between simulated and measured data. Only reference [5], which uses substrate material of low dielectric constant, is not more compact than the proposed one, and the bandwidth is also poor. So, the presented antenna is superior to all other research works listed in the table in terms of bandwidth and dielectric loss.

## 6. Conclusions

In this paper, an electric-field-coupled wideband miniaturized antenna is presented that depends on loop inductance and gap capacitance for resonance. These types of resonators can achieve compactness with low dielectric constant substrates without changing the dimension of the overall structure. In this research, techniques are adopted to improve the performance parameters of the structure. The performance of the antenna, especially the bandwidth, is enhanced by employing a defected ground structure. In addition, the gain is improved by placing parasitic elements on both sides of the microstrip feed line. The bandwidth of Antenna 1 is only 9.12%, and the gain is very low (0.063 dBi at 2.89 GHz). After applying the techniques, the bandwidth is enhanced by 116.12% and the gain is increased by 1.1 dBi. The antenna covers almost the entire 3GPP bands n77 and n78 of 5G. Because of its wideband property with compact structure and good radiation property, it is suitable for 5G embedded applications.

## Figures and Tables

**Table 1:** Detailed dimensions of the proposed antenna.

Parameters	Values (mm)	Parameters	Values (mm)
L_1_	23	W_2_	19
L_2_	13	W_3_	12
L_3_	08	W_4_	7.0
L_4_	09	W_5_	4.6
L_5_	6.5	W_6_	5.0
L_6_	3.0	W_7_	2.5
L_7_	0.6	W_8_	1.52
W_1_	23		

**Table 2:** Comparison of various compact patch antennas.

References	Substrate ε_r_	Antenna Size (Width mm × Length mm × Height mm) (λ_L_ at the Lowest Frequency)	Impedance Bandwidth (%)	Maximum Gain (dBi)
[2]	3.55	40 × 40 (height not mentioned) (0.32 λ_L_ × 0.32 λ_L_)	2.44	1
[3]	4.4	25 × 12.2 ×1.6 (0.1 λ_L_ × 0.2 λ_L_ × 0.013 λ_L_)	38.19 38.75 8.64	4.98 1.06 2.17
[5]	4.4	50 × 50 × 0.6 (0.26 λ_L_ × 0.26 λ_L_ × 0.003 λ_L_)	3.15	3.13
[6]	2.2	39 × 39 × 0.5 (0.31 λ_L_ × 0.31 λ_L_ × 0.004 λ_L)_	7.3	2.06
[7]	4.4	32 × 40 × 1.6 (0.2 λ_L_ × 0.25 λ_L_ × 0.01 λ_L)_	10.64 7.4 15.62 40	1.6 2.15 2.75 3.8
[8]	10.2	6 mm × 7 mm × 0.5 mm (0.018 λ_L_ × 0.021 λ_L_ × 0.002 λ_L_)	8.79 8.15 7.45	−26.4 −23 −20.47
[13]	4.4	40 × 40 × 0.8 (0.26 λ_L_ × 0.26 λ_L_ × 0.005 λ_L_)	22.42 5.83 6.97 11.76 4.14 17.92	1.5 6.5 1.75 2.88 4.05 2.38
[14]	4.4	20 × 20 × 0.5 (0.11 λ_L_ × 0.11 λ_L_ × 0.003 λ_L_)	2.35 2.69 1.87 4.76	0.74 0.25 0.31 2.47
[15]	4.4	19.18 × 22.64 × 1.6 (0.12 λ_L_ × 0.15 λ_L_ × 0.01 λ_L)_	2.55 3.12 8	1.36 1.57 1.83
[16]	4.4	22 × 24 × 1.59 (0.18 λ_L_ × 0.19 λ_L_ × 0.013 λ_L_)	6.84 8.97	3.02 3.26
[17]	4.4	24.8 × 30 × 1.6 (0.19 λ_L_ × 0.23 λ_L_ × 0.012 λ_L_)	6.87 5.71 9.17 5.38 5.42	2.3 2.2 2.8 2.9 3.3
Proposed Work	2.2	23 × 23 × 1.52 (0.26 λ_L_ × 0.26 λ_L_ × 0.02 λ_L_)	19.71	1.16

## Data Availability

Not applicable.
